# Exploiting adversarial style for generalized and robust weed segmentation in rice paddy field

**DOI:** 10.3389/fpls.2025.1703811

**Published:** 2025-12-01

**Authors:** Yaoxuan Zhang, Hao Cai, Jiahui Ye, Fubin Pan, Shuanglong Wu, Bob Zhang, Long Qi, Ruijun Ma

**Affiliations:** 1 College of Engineering, South China Agricultural University, Guangzhou, China; 2 Department of Computer and Information Science, University of Macau, Macao, Macao SAR, China; 3 College of Water Conservancy and Civil Engineering, South China Agricultural University, Guangzhou, China; 4 Guangdong Engineering Technology Research Center of Rice Transplanting Mechanical Equipment, Guangzhou, China; 5 State Key Laboratory of Agricultural Equipment Technology, Guangzhou, China; 6 Department of Biosystems Engineering, University of Manitoba, Winnipeg, MB, Canada

**Keywords:** weed detection, precision agriculture, precision weeding, style transfer, rice seedlings

## Abstract

In precision agriculture, effective weed management is pivotal for enhancing rice cultivation yield and quality. However, accurately differentiating weeds from rice crops remains a fundamental challenge for precision weeding. This study introduces an innovative deep-learning methodology based on style transfer for weed identification and segmentation in paddy fields. We introduce a Style-guided Weed Instance Segmentation (SWIS) method that integrates a Random Adaptive Instance Normalization (RAIN) module for stochastic style transformation and a Dynamic Gradient Back-propagation (DGB) module for adversarial feature optimization. Specifically, the RAIN module aligns feature distributions between laboratory and field environments through stochastic style transformation, enhancing cross-environment generalization. The DGB module employs adversarial optimization with gradient-guided perturbations to enhance feature robustness under complex field conditions. Experimental results demonstrate that our method achieves a Weed Intersection over Union (Weed IoU) of 70.49% on field data, significantly outperforming comparison methods. Therefore, this approach proves effective for real-world applications. Beyond its immediate applications, this research advances computer vision integration in agriculture and establishes a robust foundation for developing more sophisticated, versatile weed recognition models.

## 1 Introduction

Rice paddy weed infestation is one of the leading causes of yield reduction, quality decline, and farmland abandonment. The diversity of weed species in paddy fields poses a major challenge for effective removal. These weeds possess strong reproductive capabilities and negatively affect rice yield both directly and indirectly by competing for nutrients, inducing crop diseases, and harboring pests and pathogens Anastasiou et al. (2023). Statistics indicate that annual rice production losses due to weeds exceed 20% Baker (1974). Therefore, effective paddy weed management is crucial for ensuring stable rice production.

Mechanical and chemical weeding are the most common control methods Jin et al. (2025). However, traditional mechanical weeding lacks intelligent guidance and relies solely on root system differences between weeds and crops, often resulting in high seedling injury rates Liu et al. (2023). Chemical spraying cannot distinguish crops from weeds, leading to problems such as herbicide resistance Casanova et al. (2025), crop damage Malkani et al. (2024), and environmental pollution Jablonowski et al. (2011); Jowa and Howd (2011); Singh et al. (2018). As such, identifying weed species and obtaining precise location information is a fundamental prerequisite for enabling targeted precision operations by weeding equipment Jablonowski et al. (2011), and is thus essential for ensuring rice production security.

Computer vision provides promising solutions through spectral imaging Louargant et al. (2018); Su et al. (2025), 3D reconstruction Badhan et al. (2021), and thermal analysis Etienne and Saraswat (2019). However, the high cost of specialized sensors limits their widespread field deployment Zhang et al. (2020). By contrast, RGB camera-based systems offer a cost-effective alternative. Early computer vision methods relied on handcrafted features and traditional classifiers Bakhshipour and Jafari (2018); Islam et al. (2021); Le et al. (2019). Despite encouraging results, their performance is severely hampered by the complex backgrounds of paddy fields Mei et al. (2025); Abdalla et al. (2019), and their reliance on manual, labor-intensive feature engineering underscores the urgent need for more robust and generalizable algorithms.

Deep learning–based convolutional neural networks (CNNs) have consistently outperformed traditional methods in vision tasks by automatically extracting hierarchical features from large datasets Ma et al. (2024). They have demonstrated high accuracy across diverse domains, including astronomy Smith and Geach (2022), geology Wu et al. (2023), and medicine Ching et al. (2018). By eliminating manual feature engineering, CNNs can autonomously learn robust representations, supporting automated decision-making systems. Inspired by these advances, many researchers have applied deep learning models to agricultural monitoring and weed recognition.

Fully supervised deep learning models have set new benchmarks for weed detection accuracy in controlled settings. For instance, Osorio et al. Osorio et al. (2020) showed YOLOv3 and Mask R-CNN outperforming SVMs (94% vs. 88% F1-score) in lettuce fields; Punithavathi et al. Punithavathi et al. (2023) optimized Faster R-CNN with metaheuristics to reach 98.94% F-score; and Zou et al. Zou et al. (2021) improved U-Net for real-time segmentation, achieving 92.91% Intersection over Union (IoU). However, the exceptional performance of these models is heavily reliant on large-scale, meticulously annotated datasets, which are costly and time-consuming to acquire in agricultural contexts. More critically, even when trained on sufficient data, these models often suffer from significant performance degradation when deployed in unseen field environments with varying conditions Bertoglio et al. (2023), severely limiting their practical deployability.

To alleviate the dependency on full annotations, weakly and semi-supervised learning paradigms have gained traction. These approaches aim to achieve performance comparable to fully supervised methods using cheaper, sparser annotations (e.g., image-level labels, points, bounding boxes). Picon Picon et al. (2022) developed a semi-supervised dual network combining classification and segmentation, reaching 75.71% balanced accuracy for seven weed species with reduced annotation needs. Chen et al. Chen et al. (2024b) introduced PIS-Net, a point-supervised instance segmentation network for rice weeds, where sparse point labels achieved average precision (AP) 38.5 and AP50 68.3—close to 90% of fully supervised performance. Despite their success in reducing annotation costs, these methods often lack robustness under changing field conditions and may require partial relabeling for new environments Luo et al. (2020), which conflicts with the low-latency, low-cost requirements of agricultural operations.

In parallel, Generative Adversarial Networks (GANs) and their variants have been explored to tackle the critical issue of domain generalization. The core idea is to use image-to-image translation to augment training data diversity by transferring the style of source domain images to mimic diverse target domain conditions (e.g., different soil types, lighting) while preserving semantic content. Studies such as Espejo-Garcia et al. (2021) have demonstrated the potential of GANs for data augmentation, with Espejo-Garcia et al. achieving a 99.07% F1-score for tomato weeds using DCGAN-augmented transfer learning. However, existing GAN-based approaches face two key limitations: first, they typically rely on an offline, two-stage ‘train-translate-retrain’ pipeline, preventing real-time model adaptation; second, their style transformation often lacks controlled randomness and differentiability, making it difficult to systematically and realistically model complex, continuous environmental variations like soil reflectance and illumination gradients. Therefore, developing a new mechanism capable of real-time, controllable, and differentiable simulation of environmental dynamics during training remains an urgent need.

In this study, we make the following contributions:

- We propose RAIN, a stochastic style exploration mechanism that enables real-time adaptation to agricultural field dynamics through differentiable modeling of soil reflectance and illumination gradients, thereby enhancing cross-environment generalization.
- We establish a closed-loop DGB framework that drives robust feature optimization across diverse paddy environments, ensuring model stability under complex field conditions via self-regulating feedback.
- We develop the SWIS framework that achieves high-precision crop–weed segmentation under varying field environments with single-annotation effort, substantially reducing deployment costs while maintaining robustness.

## 2 Materials and methods

### 2.1 Weed data collection

**Figure 1:**
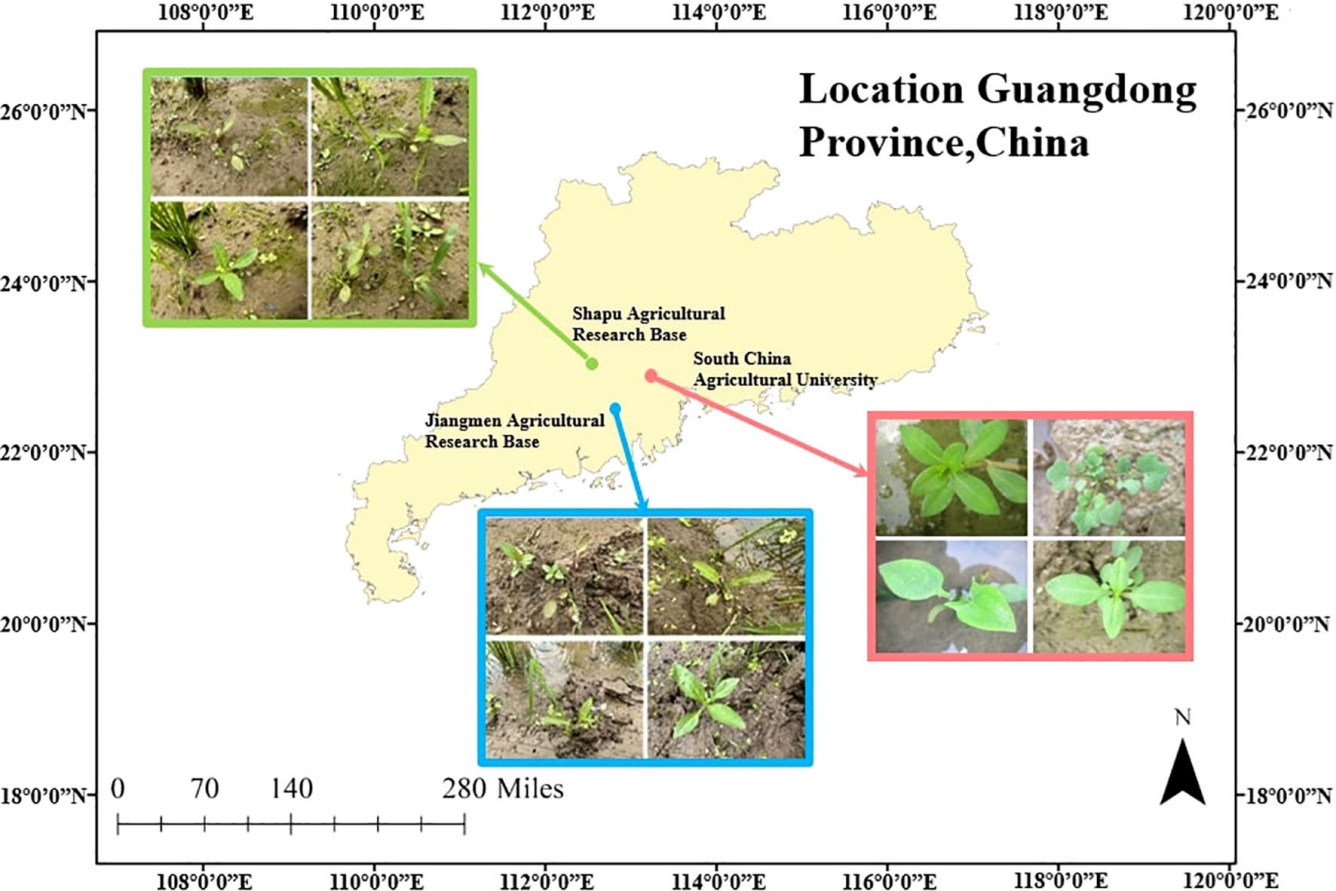
Experimental data collection location.

As illustrated in **Figure 1**, we collected paddy weed data in Guangdong Province, China. We constructed a bespoke paddy-weed dataset composed of two complementary subsets: a *laboratory subset* recorded under controlled conditions and a *field subset* captured in real paddy fields. These two subsets were designed to evaluate the proposed model. The dataset includes six weed species: barnyard grass (*Echinochloa crus-galli*), purple nutsedge (*Cyperus rotundus*), eclipta (*Eclipta prostrata*), redroot amaranth (*Cardamine occulta*), alligator weed (*Alternanthera philoxeroides*), and water dropwort (*Oenanthe javanica*).

**Figure 2:**
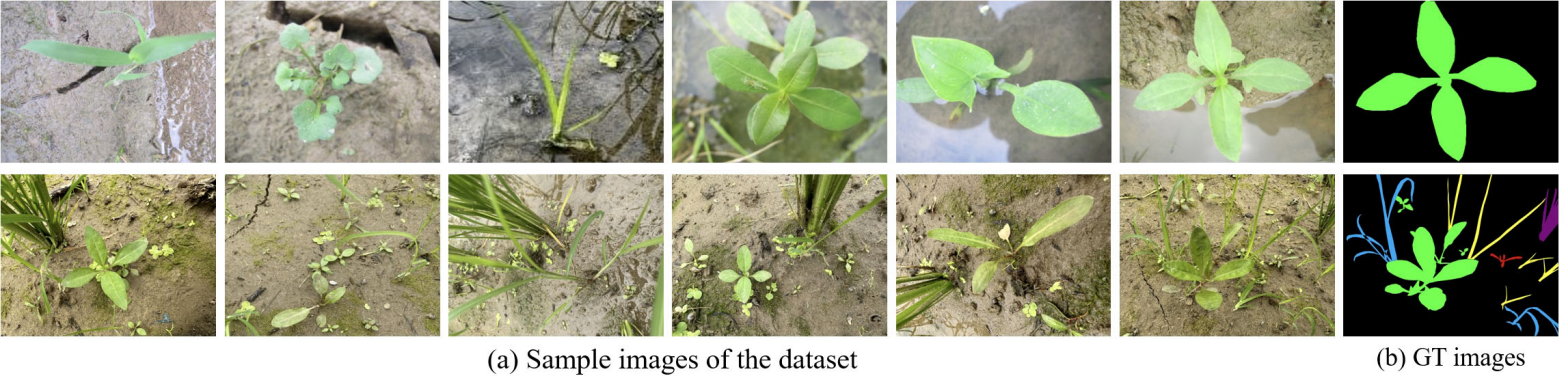
Sample images from the laboratory (first row) and field subsets (second row).

For the laboratory subset (as shown in **Figure 2**), the six species of paddy weeds were cultivated at South China Agricultural University (113°20′43′′E, 23°9′29′′N) in April and July 2023. At 25 days old, the weeds were transplanted into soil tanks within the laboratory. To simulate paddy field conditions, rice seedlings were planted at 13 cm to 15 cm intervals, with weed plants randomly interspersed among them. Images were captured from May to August 2023 using a Canon IXUS 1000 HS digital camera equipped with a 36 mm to 360 mm f/3.4–5.6 lens. The camera, mounted on a platform, was positioned 10 cm to 20 cm above the water surface. A total of 1,800 images with a resolution of 3648 × 2736 pixels were collected.

For the field subset (as shown in **Figure 2**), images were collected between April and August in the years 2022 to 2025 at two agricultural research bases in Guangdong Province: the Shapu Agricultural Research Base in Zhaoqing (112°39′46′′E, 23°8′52′′N) and the Jiangmen Agricultural Research Base (113°6′6′′E, 22°30′16′′N). Using the same camera system, a total of 2,000 images were captured from a height of 20 cm to 40 cm. This subset reflects realistic field complexity, including cluttered backgrounds, dense plant overlaps, and mixed-species growth, providing a realistic and challenging benchmark for segmentation under operational paddy filed scenarios.

The laboratory subset contains a total of 1800 annotated weed instances and 263 annotated paddy instances, with individual class counts ranging from 220 to 360 instances. The field subset includes 10389 instances with more varied distribution reflecting natural occurrence patterns, ranging from 128 to 3746 instances per species. Instance sizes vary from 15×15 pixels for small weeds to 250×300 pixels for larger specimens, capturing the natural size variability encountered in real-field conditions.

For data preprocessing, we implemented a comprehensive pipeline to transform raw images into the training dataset. All images were first normalized using ImageNet mean and standard deviation values. We then applied random augmentations including horizontal flipping (probability=0.5), random rotation 120 (± 15°), and color jittering (brightness=0.2, contrast=0.2, saturation=0.2, hue=0.1) to improve model robustness. To handle class imbalance, we employed stratified sampling during training.

The field dataset was rigorously partitioned to ensure unbiased evaluation: 10% (200 images) served as a validation set for hyperparameter tuning, while the remaining 90% (1,800 images) constituted the test set for all reported performance metrics.

All images were annotated at the pixel level with instance-wise semantic labels using LabelMe to produce ground-truth (GT) masks for each species. To reduce training complexity and mitigate GPU memory constraints, we downsampled every image to 640 × 480 pixels to form the final dataset used in this study.

### 2.2 Architecture overview

**Figure 3:**
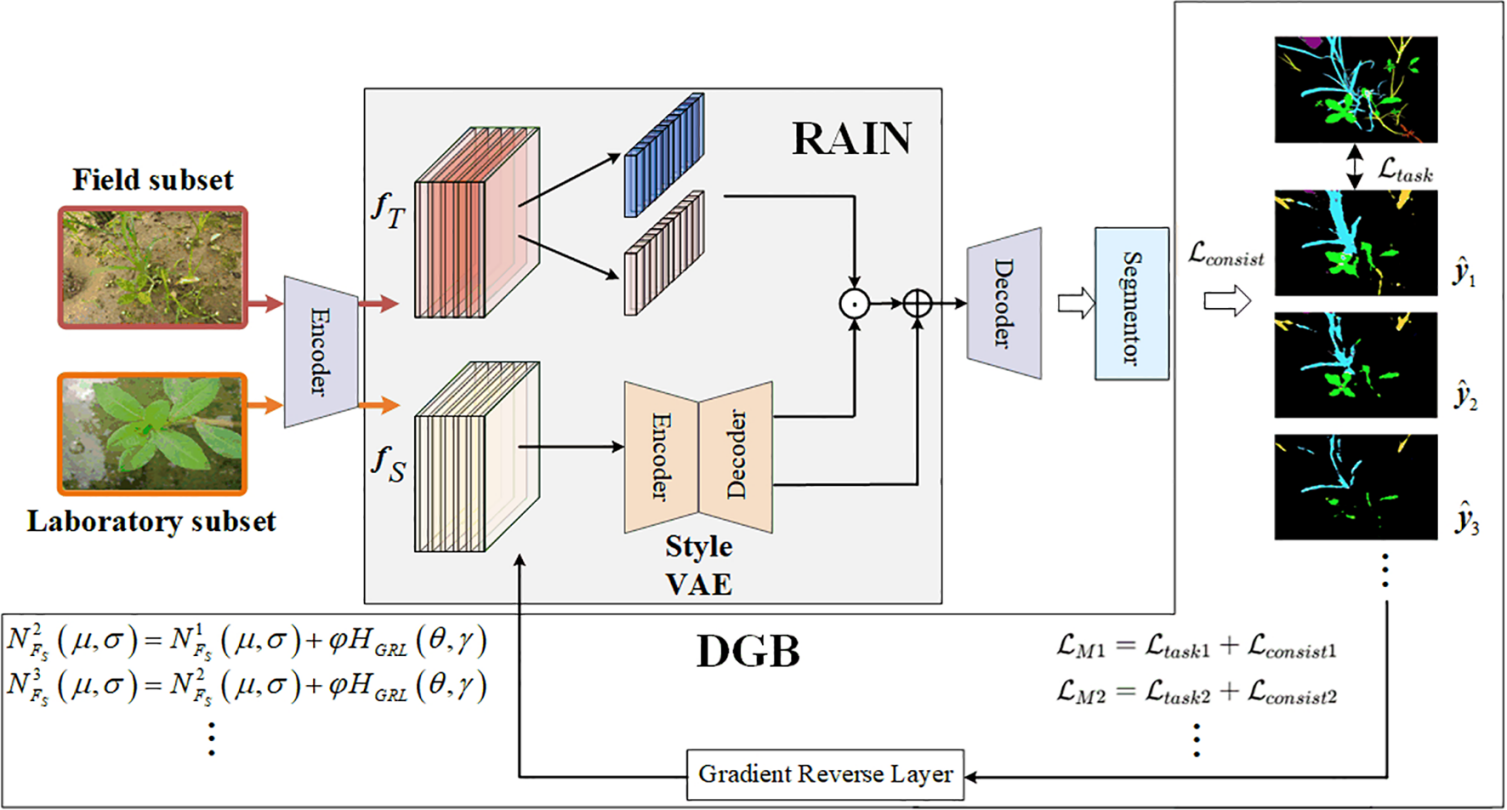
Architecture of SWIS.

**Figure 3** shows the overall framework of the SWIS framework. This framework comprises three main components: the RAIN module, the instance segmentation module, and the DGB module.

The RAIN module narrows the style distribution gap between laboratory subset and field subsets by adaptively modulating the feature statistics of weed imagery through stochastic instance normalization. The DGB module implements an adaptive adversarial optimization mechanism: by injecting gradient perturbations during backpropagation, driven by segmentation-loss feedback, DGB encourages the RAIN module to learn style representations that better reflect field conditions, thereby improving generalization for instance segmentation.

The core idea is that DGB uses the segmentation loss as a perturbation signal and applies gradient-aligned perturbations, where style parameters are updated along the direction of the cost function gradient. This constraint reduces the negative impact of style perturbations on semantically discriminative features. The closed-loop scheme progressively enhances cross-environment generalization while preserving critical semantic cues for accurate segmentation. In the following subsections we detail the RAIN and DGB components.

### 2.3 Random adaptive instance normalization

**Figure 4:**
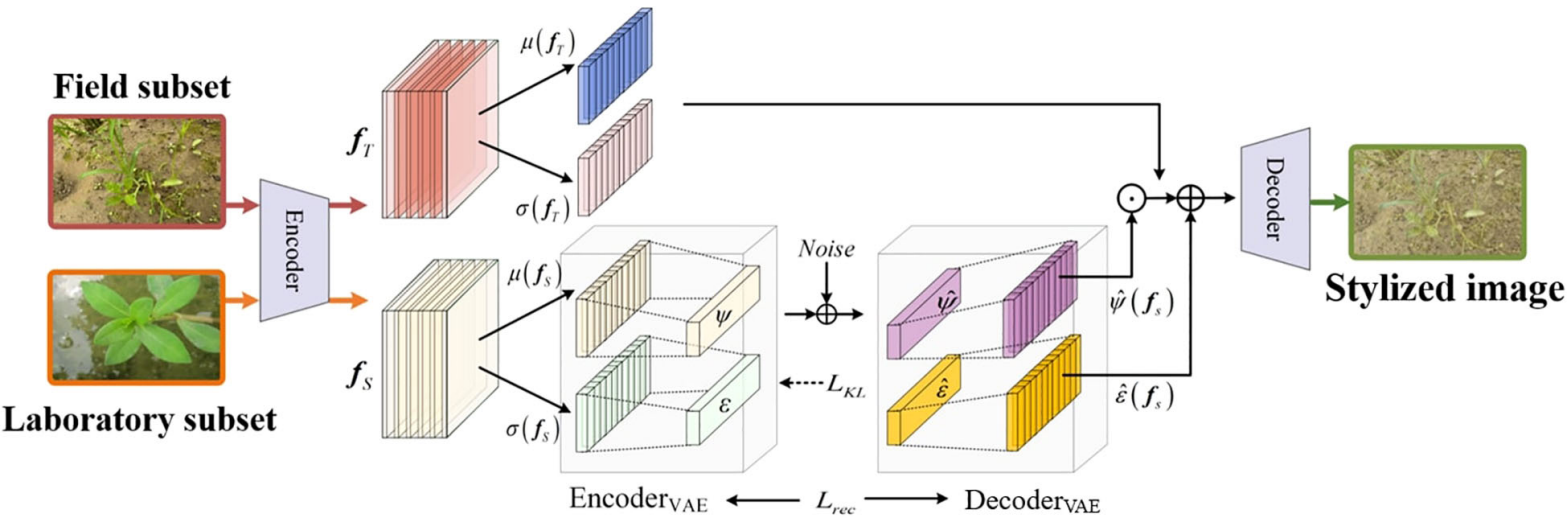
Architecture of RAIN.

As visualized in **Figure 4**, the RAIN module is designed to enhance the model’s adaptability to the field subset. By adaptively aligning the feature distributions between the laboratory and field subsets, the module effectively reduces their domain gap, significantly enhancing the segmentation accuracy of weed instance segmentation models in the field subsets. RAIN consists of three subcomponents: a deep feature extraction network, a style encoding network, and an image generation network. Among them, the style encoding network and the image generation network together form the Style VAE, which is responsible for learning the style distribution of the field subset and performing random sampling and reconstruction in the latent space. This Style VAE is central to RAIN’s distribution-alignment and style-generalization capability.

The processing pipeline of the RAIN module consists of three stages. First, deep features are extracted from the input images of the real paddy field dataset, and their higher-order statistics are computed as style representations. This process is achieved by calculating the mean and standard deviation of the feature maps:

(1) $$s=[\mu (f_{s});\sigma (f_{s})]$$

In Equation 1, *f_s_* denotes the deep features extracted from the laboratory subset; *µ* and *σ* represent spatial mean and standard deviation across feature-map channels; and **s** constitutes the resulting style statistical vector.

Subsequently, the encoder of the Style VAE maps the style statistical vector **s** to the parameters of the latent style distribution (mean *ξ* and standard deviation *ψ*), and samples the latent code **z** from this distribution via the reparameterization trick. By introducing stochastic noise during sampling, this process enables the model to capture the style diversity inherent in real paddy field datasets, thereby enhancing the generalization capability of the weed instance segmentation model, as shown in Equation 2:

(2) $$z=\xi +\psi ⊙{Є}_{noise}$$

where *ξ* and *ψ* denote the mean and standard deviation of the style vector learned from the field subset, controlling the center and range of style transformation; ⊙ indicates element-wise multiplication; *ϵ_noise_* is a random noise vector sampled from a standard normal distribution, introducing stochastic perturbations; and **z** represents the newly generated style encoding vector.

Finally, the learned style statistics are injected into content features (from the laboratory images) via instance-normalization-based modulation:

(3) $$f_{out}=\sigma (f_{s})⊙\frac{f_{T}-\mu (f_{T})}{\sigma (f_{T})}+\mu (f_{s})$$

In Equation 3, *f_T_* represents the deep features extracted from field dataset images; *µ*(*f_T_*) and *σ*(*f_T_*) denote the mean and standard deviation computed along the spatial dimensions of the feature maps, respectively, used for instance normalization; *µ*(*f_s_*) and *σ*(*f_s_*) represent the style statistics learned from the real paddy field dataset, corresponding to the mean and standard deviation of the feature distribution; and *f_out_* is the output feature after style adaptive transformation.

Traditional weed instance segmentation methods exhibit limited generalization capability on paddy field datasets, leading to degraded segmentation accuracy. While standard style transfer methods (e.g., AdaIN) can alter image appearance, they often cause degradation of crucial discriminative features of weeds during the style transfer process, consequently compromising the accuracy of weed instance segmentation. RAIN adaptively adjusts feature statistics through learnable distribution parameters, effectively reducing the distribution discrepancy between laboratory and real paddy field datasets while significantly mitigating feature degradation caused by stylization. The module employs a multi-objective loss optimization strategy that effectively balances style adaptation and preservation of discriminative weed features. The overall loss function is defined as:

(4) $$L_{RAIN}=L_{c}+\lambda_{s}L_{s}+\lambda_{KL}L_{KL}+\lambda_{Rec}L_{Rec}$$

In Equation 4, $L_{c}$ denotes content loss that constrains the preservation of discriminative weed features in generated images during style transfer to maintain semantic consistency for segmentation purposes; $L_{s}$ represents style loss that drives the statistical distribution of generated images toward approximating the style characteristics of real paddy field datasets; $L_{KL}$ indicates the ullback–Leibler (KL) divergence loss that regularizes the latent space distribution of Style VAE to approach a standard normal distribution, thereby enhancing generalization capability; and $L_{Rec}$ denotes reconstruction loss that ensures the decodability of style representations by quantifying the discrepancy between style vectors reconstructed by the Style VAE decoder and their original counterparts. The coefficients *λ_s_*, *λ_KL_*, and *λ_Rec_* serve as weighting hyperparameters for the respective loss components.

Among these loss terms, $L_{KL}$ and $L_{Rec}$ in the aforementioned formula specifically constitute the training objectives for the Style VAE component, as shown in Equations 5 and 6, respectively:

(5) $$L_{KL}=D_{KL}(N(\xi ,\psi^{2})∥N(0,\text{I}))$$

(6) $${ℒ}_{Rec}=∥\mu (f_{s})\;⊙\;\sigma (f_{s}),\;\mu (f_{s}\hat{)\;⊙\;\sigma }\;(f_{s}){∥}_{2}$$

where N(ξ,ψ) represents the latent style distribution learned by the Style VAE encoder, where ξ denotes the mean vector of this distribution and ψ denotes the standard deviation vector of this distribution. N(0,I) represents the standard normal distribution, and $\mu (f_{s}\hat{)\;⊙}(f_{s})$ represents the reconstructed style vector.

### 2.4 Dynamic gradient back-propagation module

The DGB module enhances the generalization ability of the weed instance segmentation model via adaptive adversarial perturbations in feature space. This module processes the input feature map and first calculates the mean and variance of specific instances:

(7) $$\mu_{b,c}(x)=\frac{1}{HW}\sum_{i=1}^{HW}x_{b,c,i}$$

(8) $$\sigma_{b,c}^{2}(x)=\frac{1}{HW}\sum_{i=1}^{HW}{\left(x_{b,c,i}-\mu_{b,c}(x)\right)}^{2}$$

In Equations 7 and 8, *H* denotes height, *W* denotes width, *b* denotes batch index, *c* denotes channel index, and *i* denotes spatial position index.

This module proactively explores the feature space beyond the training distribution. This mechanism introduces an adaptive adversarial standard deviation parameter to dynamically adjust the variance of the feature map, thereby aligning the feature distribution of the laboratory subset with that of the field subset in the feature space, as shown in Equation 9:

(9) $$x_{t}=\sigma_{adv}⊙(\frac{x-\mu (x)}{\sigma (x)})+\mu_{adv}$$

where, $x_{t}$ denotes the transformed adversarial feature map, which serves as the input to the subsequent layers of the network; $\sigma_{adv}$ denotes the adversarial standard deviation, a learnable parameter used to control the degree of dispersion of the feature distribution.

**Figure 5:**
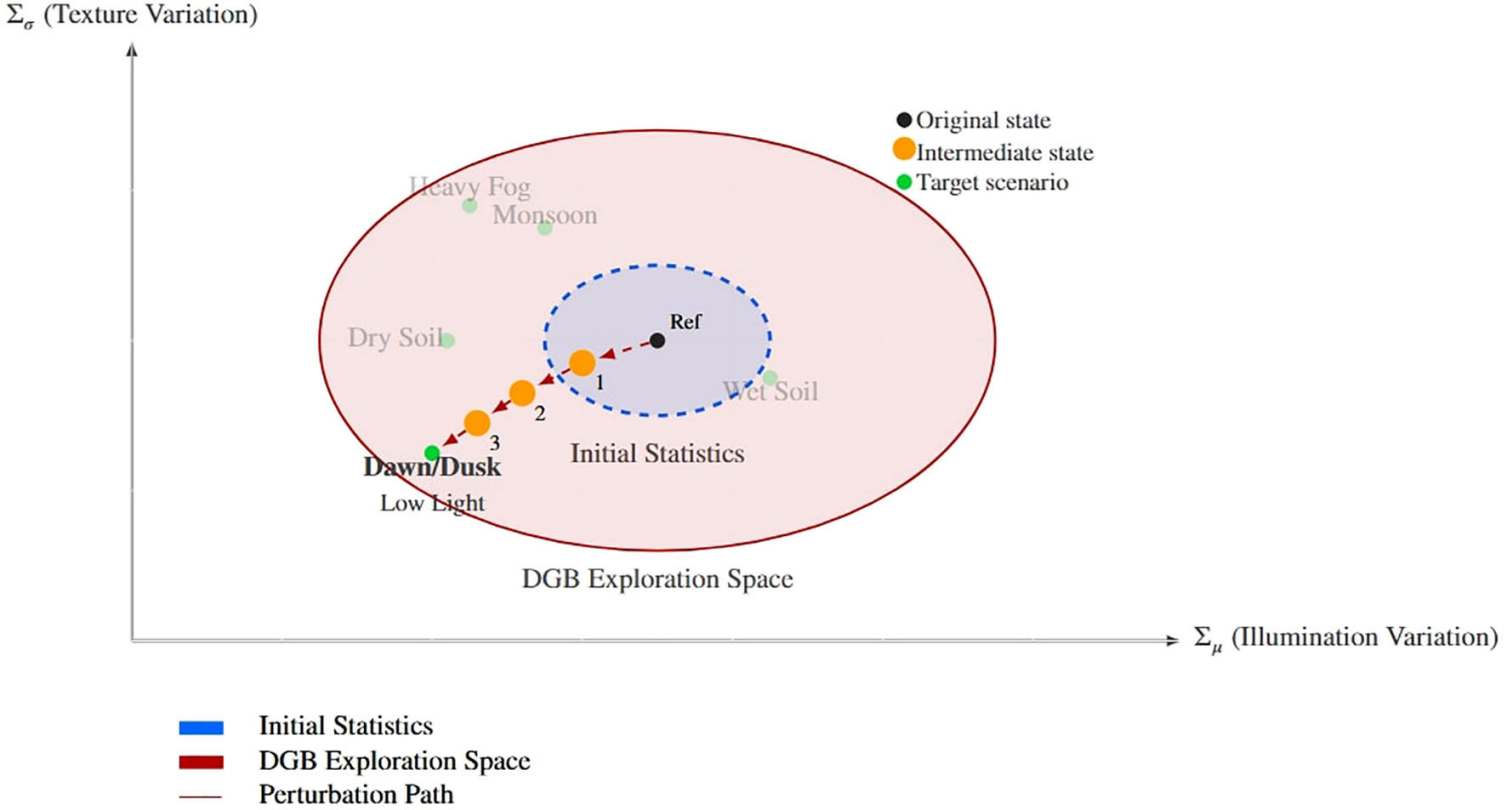
Conceptual representation of the dynamic perturbation process in rice fields. The visualization shows how the DGB module transitions from reference conditions (Ref) to target field states (Dawn/Dusk) through parameter adjustments (Σ*_µ_*, Σ*_σ_*), exploring intermediate states within the expanded feature space.

**Figure 6:**
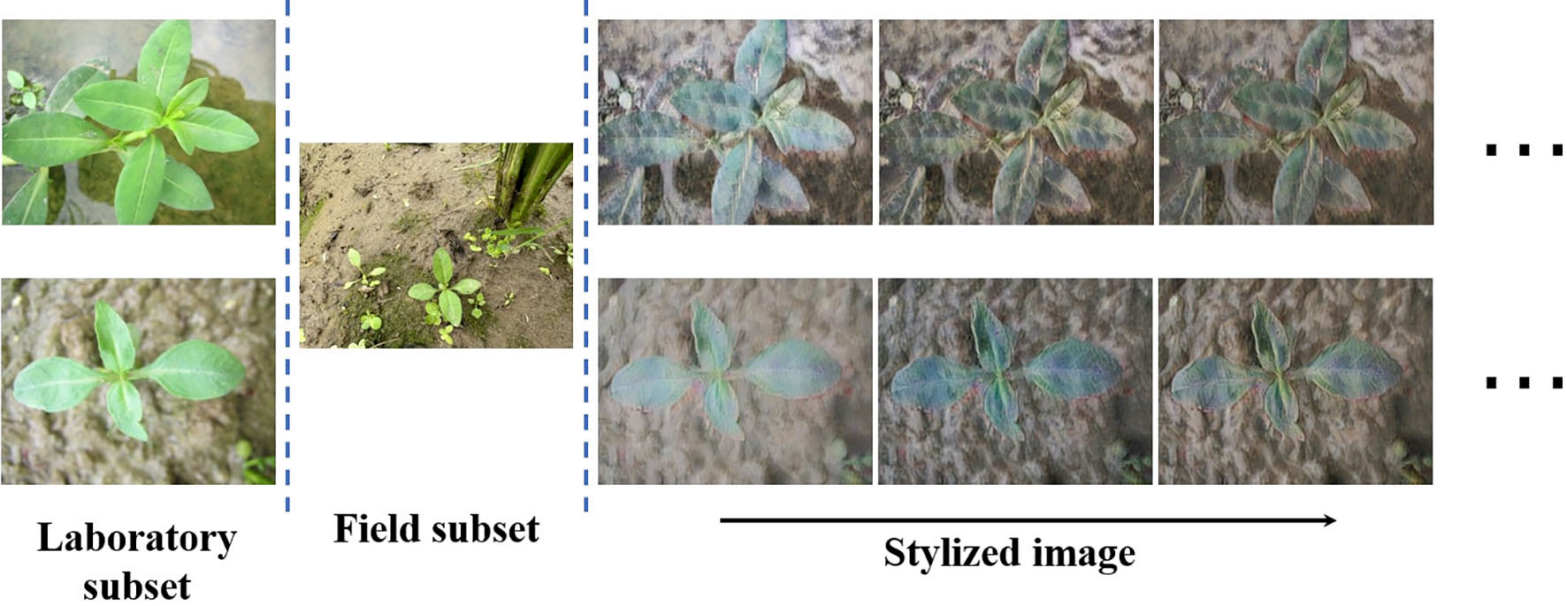
Visual comparison of style transfer.

As shown in **Figures 5** and **6**, this mechanism enables the model to exhibit strong generalization ability and achieve migration from the laboratory dataset to the real paddy field environment. The perturbation intensity parameter is automatically adjusted during the training process, and this strategy helps enhance the model’s robustness. Traditional weed instance segmentation methods suffer from poor generalization and thus struggle to meet the needs of actual field applications. DGB addresses these issues through self-adjusting adversarial training: while achieving adaptive style transfer, it ensures the consistency of weed features. The gradient reversal layer establishes a feedback loop, and the increased style diversity enhances feature robustness. This further enables accurate weed segmentation in complex environments, forming a self-reinforcing cycle:

(10) $$\frac{\partial L_{adv}}{\partial \text{Σ}}=-\lambda_{GRL}\frac{\partial L_{task}}{\partial \text{Σ}}$$

In Equation 10, $\frac{\partial L_{adv}}{\partial \text{Σ}}$ represents the gradient of the adversarial loss with respect to the transformation parameters, $\frac{\partial L_{task}}{\partial \text{Σ}}$ represents the gradient of the weed instance segmentation loss with respect to the transformation parameters, and $\lambda_{GRL}$ denotes the gradient reversal coefficient.

(11) $$N_{F_{s}}^{i+1}(\mu ,\sigma )=N_{F_{s}}^{i}(\mu ,\sigma )+\varphi H_{GRL}(\theta ,\gamma )$$

In Equation 11, *i* denotes the iteration index during training, *N_Fs_* represents the Gaussian distribution of image style features, *φ* controls the update step size for distribution adjustment, *H_GRL_* indicates the Gradient Reversal Layer, *θ* regulates the strength of gradient flow, *γ* controls the direction of gradient propagation.

The model can generate more diverse and complex feature distributions during iterative network training by leveraging the feedback from gradient backpropagation. The consistency loss term further stabilizes feature representation amid style variations, thereby ensuring the robustness of the weed instance segmentation model, as shown in Equation 12:

(12) $$L_{consist}=\frac{1}{N}\sum_{i=1}^{N}|z_{i}-\bar{z}{|}_{2}$$

### 2.5 Loss function

The loss function of the SWIS model balances the feature extraction ability with the generalization ability of the weed instance segmentation model. The supervised task loss utilizes labeled weed images and their real labels to supervise the model in learning weed features. Segmentation errors will drive parameter adjustment:

(13) $$L_{task}=\ell (M(x_{S}),y_{S})$$

In Equation 13, $M(x_{S})$ represents the predicted output result of the weed image $x_{S}$, and $y_{S}$ represents the true annotation.

The consistency loss improves instance segmentation accuracy and enhances model generalization by constraining the model’s response to environmental changes between two subsets:

(14) $$L_{consist}=\frac{1}{N}\sum_{i=1}^{N}∥z_{i}-\bar{z}{∥}_{2}$$

In Equation 14, $z_{i}$ represents the feature of a single image, and $\bar{z}$ refers to the batch mean.

The combined objective function ensures dual capabilities: the task loss addresses the robustness of accurate weed instance segmentation, while the consistency loss improves the generalization ability of the segmentation model. Through joint optimization of the two loss functions, the model achieves reliable segmentation performance on field subset, as shown in Equation 15:

(15) $$L_{M}=L_{task}+\lambda L_{consist}$$

The specific training process can be referred to - **Algorithm 1**. The implementation parameters are set as follows: iteration depth *n* = 20*k*, model learning rate *α* = 0.03, style perturbation rate *β* = 0.01, consistency weight *λ* = 0.8, and batch size 64.

Algorithm 1

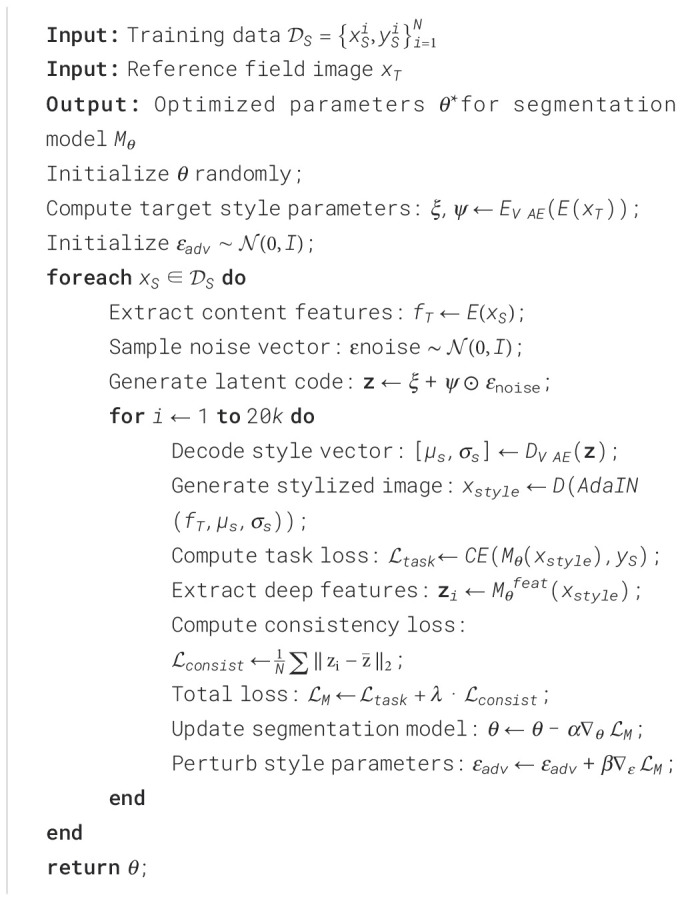

## 3 Experiments

### 3.1 Implementation details

All experiments, including baseline reimplementations, were conducted under identical conditions using PyTorch Paszke et al. (2017) on a single NVIDIA RTX-3090 GPU (24GB VRAM) to ensure fair comparison. To maintain consistency, all models followed the same training procedures, including optimizer choices, learning rates, and data preprocessing pipelines. We employed the DeepLabv3+ architecture with a ResNet-50 backbone for the instance-segmentation network. The training protocol consisted of two sequential phases. First, the RAIN module was pre-trained on a combined set of source and stylized images using the Adam optimizer Kingma and Ba (2014). Second, with the RAIN parameters frozen, we trained the instance segmentation model inside the SWIS framework using SGD Bottou (2010). Optimal hyperparameters—selected via grid search—were set as follows: *λ* = 2 × 10^−4^ (content weight), *λ_s_* = 1.0 (style weight), *λ_k_* = 1.0 (KL divergence weight), and *λ_r_* = 5.0 (reconstruction weight).

### 3.2 Evaluation metrics

We evaluate segmentation performance using four commonly used, pixel-level metrics: mIoU (Mean Intersection over Union), Precision, Recall, and F1-score. mIoU measures the average overlap between predicted and ground-truth regions across all classes. Precision indicates the fraction of predicted positive pixels that are correct, while Recall measures the fraction of true positive pixels that are recovered by the model. The F1-score is the harmonic mean of Precision and Recall and balances the two. Higher values for all metrics indicate better performance (range [0,1]). The formulas for these metrics are provided below:

(16) $$\text{mIoU}=\frac{1}{C}\sum_{c=1}^{C}\frac{TP_{c}}{TP_{c}+FP_{c}+FN_{c}}$$

(17) $$\text{Precision}=\frac{TP}{TP+FP}$$

(18) $$\text{Recall}=\frac{TP}{TP+FN}$$

(19) $$\text{F}1-\text{score}=2\times \frac{Precision\times Recall}{Precision}+Recall$$

In Equations 16–19, *TP_c_*, *FP_c_* and *FN_c_* are the true positive, false positive, and false negative pixel counts for class *c*; *TP*, *FP* and *FN* without subscripts denote the corresponding totals used for global (pixel-level) Precision/Recall/F1-score. In this work we report mIoU averaged across classes, and Precision/Recall/F1 score computed at the pixel level (micro-averaged across classes). True Positives, False Positives, and False Negatives were all computed at the pixel level to calculate the above metrics.

### 3.3 Comparisons with different segmentation models

#### 3.3.1 Comparison of different segmentation models on the laboratory subset

**Figure 7:**
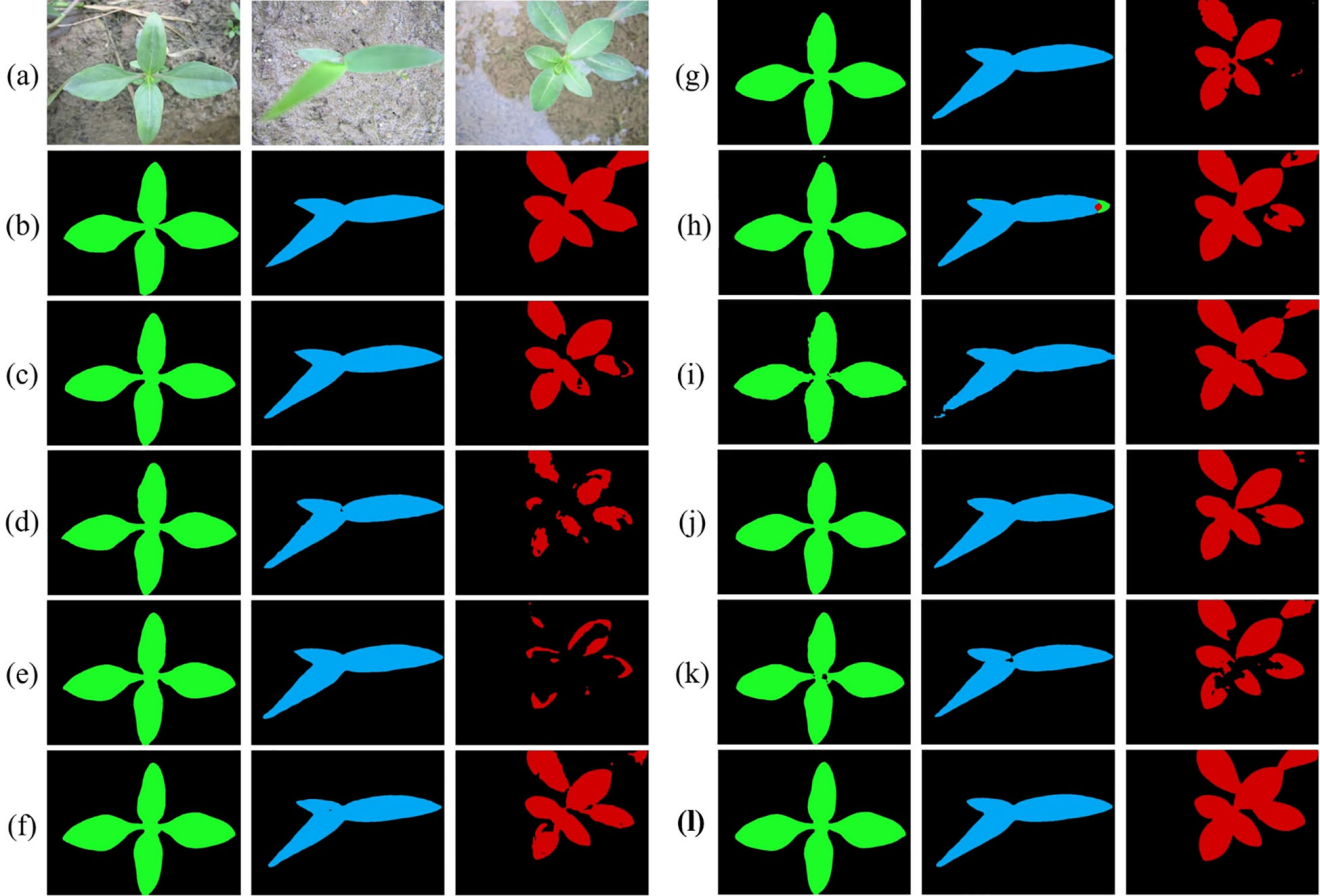
Comparative segmentation results of ten models on source-only dataset. Color coding: green = Eclipta prostrata, blue = Barnyard grass, red = Alligator Weed, black = background. **(a)** Input images; **(b)** Ground truth (GT); **(c)** HGFormer; **(d)** SAN; **(e)** WS-SIS; **(f)** PMNet; **(g)** ESL; **(h)** EDAPS; **(i)** PSANet; **(j)** PIDNet; **(k)** DSCA-PSPNet; (l) Ours.

To verify model performance in a controlled setting, we evaluated the SWIS framework on the laboratory subset by splitting the data into training and test sets at an 8:2 ratio. The results are reported in **Table 1** and shown in **Figure 7**. When training and test images are acquired under the same conditions, all compared methods achieve reasonable segmentation performance. Even so, the proposed method retains a clear advantage: it attains mIoU=90.54%, Precision=96.67%, and F1-score=91.60%, with all three core metrics being the best among the methods compared. In particular, our mIoU of 90.54% exceeds the second-best PMNet (87.28%) by 3.26 percentage points and outperforms PIDNet (83.05%) by 7.49 percentage points, indicating substantially stronger overall segmentation accuracy.

**Table 1:** Evaluation index scores of ten models on laboratory subset.

Method	mIoU	Prec.	Rec.	F1
	(%)	(%)	(%)	(%)
HGFormer Ding et al. (2023)	79.89	84.18	87.77	85.90
SAN Xu et al. (2023b)	82.66	87.90	89.46	89.80
WS-SIS Kim et al. (2023)	77.17	83.15	84.54	83.84
PMNet Chen et al. (2024a)	87.28	92.26	90.92	91.55
ESL Ma et al. (2023)	81.50	83.77	90.29	87.04
EDAPS Saha et al. (2023)	81.65	83.98	90.73	87.25
PSANet Li et al. (2024)	78.27	81.47	88.54	84.88
PIDNet Xu et al. (2023a)	83.05	86.50	91.69	89.10
DSCA-PSPNet Yuan et al. (2024)	80.89	83.54	90.13	86.70
Ours	90.54	96.67	87.38	91.60

We emphasize that these gains—observed under controlled laboratory conditions—reflect improved robustness of our approach. The RAIN module standardizes and reconstructs weed appearances, which strengthens feature extraction for weed regions, while the DGB module promotes consistency of weed characteristics across samples. Together, these mechanisms enable more accurate and stable segmentation in the laboratory setting.

#### 3.3.2 Comparison of different segmentation models on the field subset

**Figure 8:**
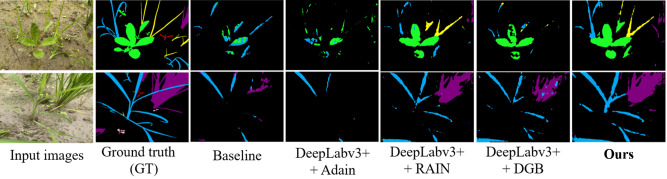
Segmentation performance comparison on laboratory subset. Color coding: purple = crop, blue = barnyard grass, yellow = purple nutsedge, green = eclipta, pink = redroot amaranth, red = alligator weed, black = background.

We evaluated generalization by training models on the laboratory subset and testing them on the unseen field subset. As shown in **Figure 8**, under this cross-domain setting, our method achieved mIoU=39.66%, substantially outperforming nine state-of-the-art segmentation models (**Table 2**). This corresponds to a relative improvement of approximately 58.4% over the best comparator, DSCA-PSPNet (25.04%), and an absolute increase of 14.62 percentage points.

**Table 2:** Evaluation index scores of ten models on field subset.

Method	Weed IoU (%)	mIoU (%)	Prec. (%)	Rec. (%)	F1 (%)
HGFormer	37.71	24.52	57.37	44.77	50.42
SAN	35.24	21.25	82.94	36.29	50.93
WS-SIS	43.60	23.18	85.04	41.30	55.14
PMNet	41.41	24.06	84.23	43.60	57.47
ESL	37.41	20.92	23.54	37.46	29.03
EDAPS	33.96	21.59	58.65	42.00	49.17
PSANet	48.75	21.83	64.68	37.95	48.02
PIDNet	42.87	21.66	61.54	40.07	48.51
DSCA-PSPNet	53.94	25.04	**93.37**	42.86	58.97
Ours	**70.49**	**39.66**	71.77	**74.08**	**72.91**

The best results are highlighted in bold.

The model also demonstrated a strong precision–recall balance: Precision=71.77%, Recall =74.08%, and F1-score =72.91%. The F1-score represents an absolute gain of 15.44 percentage points (≈26.9% relative improvement) compared with the closest competitor, PMNet (57.47%).

From a practical agricultural perspective, the Weed IoU metric provides crucial insights into the model’s field applicability. Our method achieves a remarkable 70.49% Weed IoU, significantly outperforming all competing approaches. This indicates that when considering weeds as a unified category—which aligns with actual weeding operations, our model demonstrates superior performance which makes the model directly suitable for deployment in real-world field scouting and precision weed management. The balanced Precision (71.77%) and Recall (74.08%) values further confirm the model’s reliability: it correctly identifies most actual weed regions while maintaining low false positive rates. This balance is essential for practical deployment, as high Precision minimizes herbicide waste, while high Recall ensures effective weed control.

**Figure 9:**
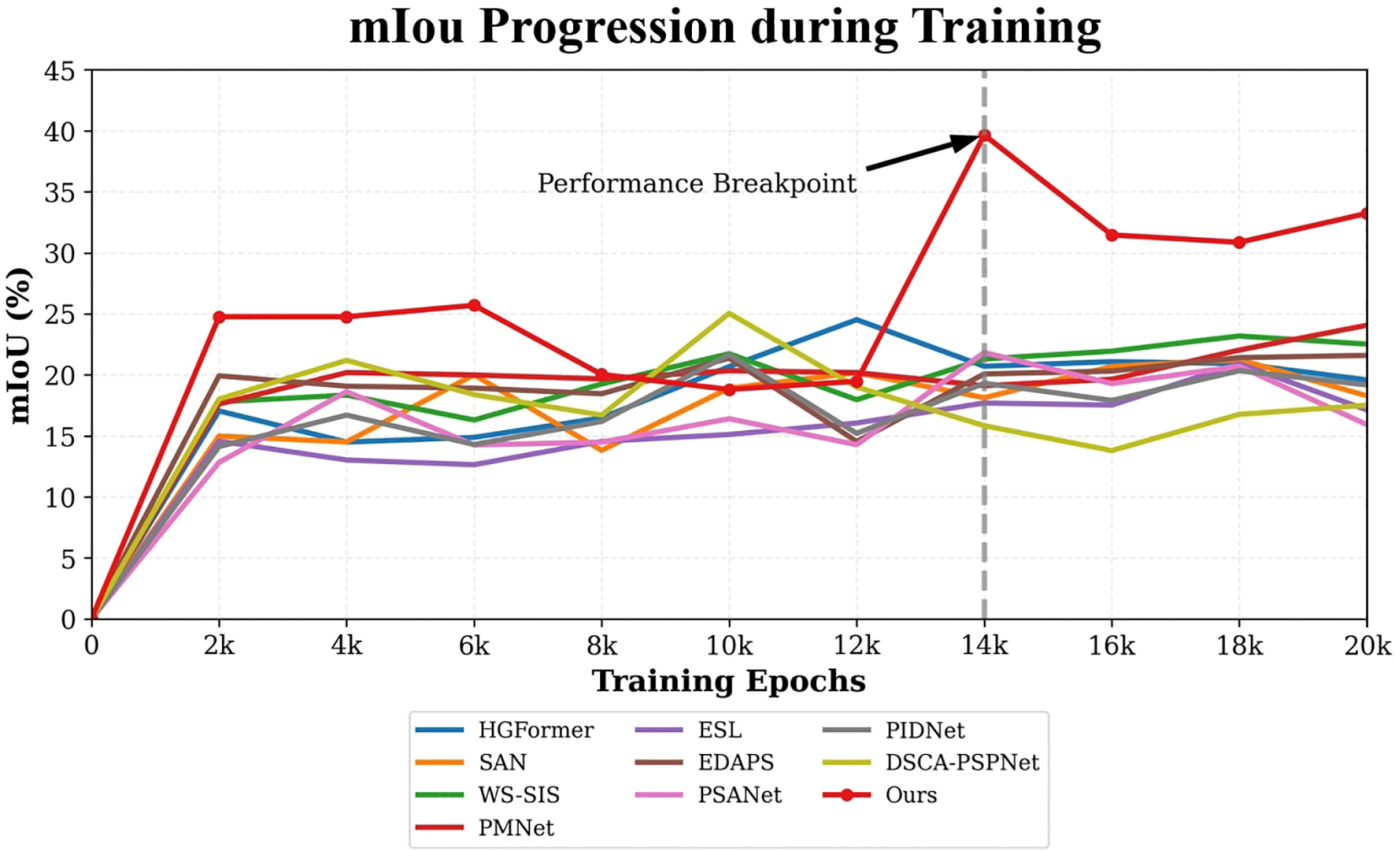
mIoU progression during field adaptation training.

**Figure 10:**
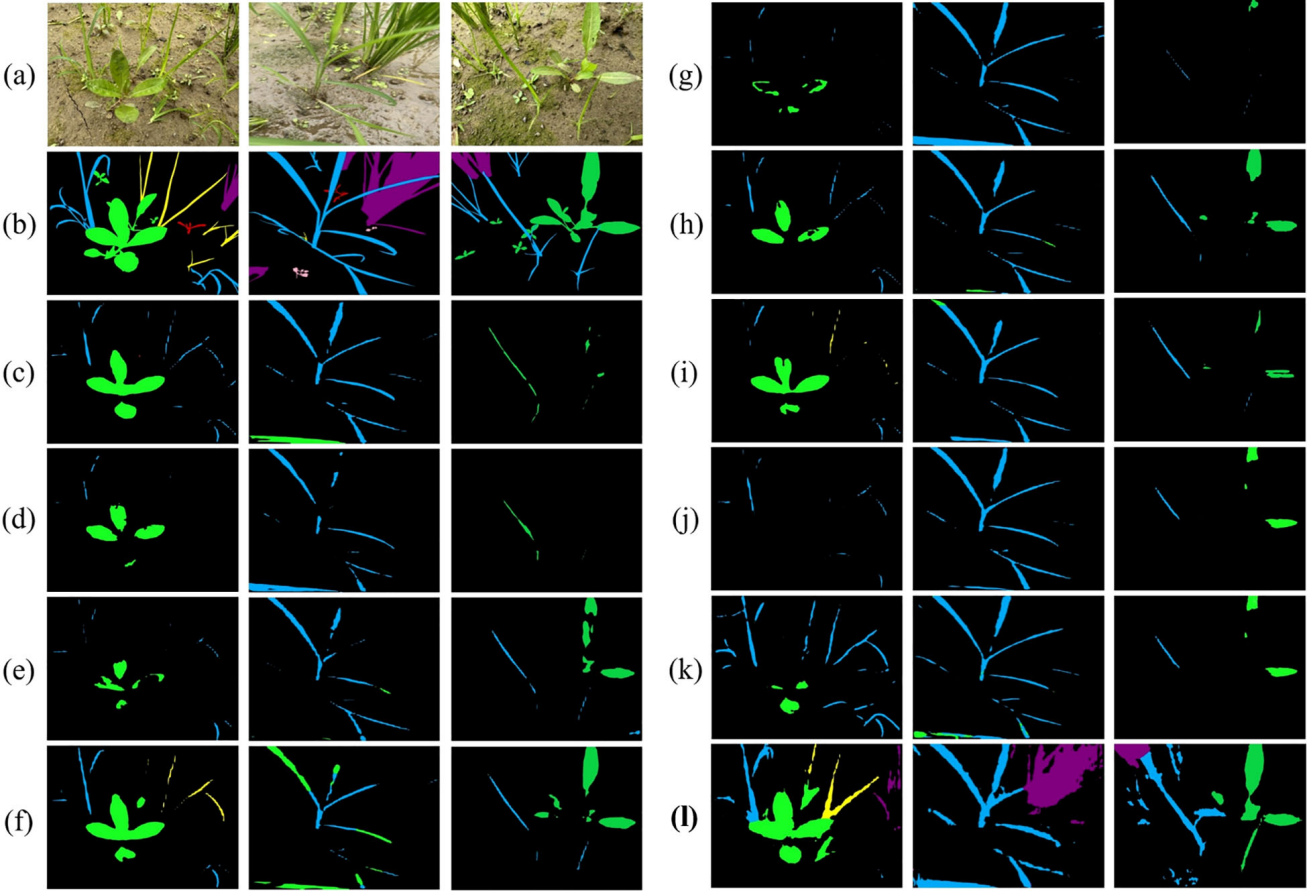
Qualitative results on operational field data. Color coding: green = eclipta, blue = barnyard grass, yellow = purple nutsedge, pink = redroot amaranth, red = alligator weed, black = background. **(a)** Input; **(b)** Ground truth; **(c)** HGFormer; **(d)** SAN; **(e)** WS-SIS; **(f)** PMNet; **(g)** ESL; **(h)** EDAPS; **(i)** PSANet; **(j)** PIDNet; **(k)** DSCA-PSPNet; **(l)** Ours.

Analysis of training dynamics (**Figure 9**) shows that our model reached peak performance (39.66% mIoU) at around 14,000 epochs and maintained stable performance above 30% mIoU thereafter. In contrast, comparative methods tended to plateau below 25% mIoU and exhibited larger fluctuations. Qualitative results (**Figure 10**) corroborate these quantitative findings: segmentation maps produced by our approach more closely match the ground-truth annotations, show consistent performance across diverse weed species, and exhibit fewer misclassification errors.

The performance advantage of SWIS over the best baseline, DSCA-PSPNet, was statistically validated via a paired t-test. With a mIoU of 39.66% (± 0.91) versus 25.04% (± 1.81) for the baseline, the observed improvement is highly significant (*p<*0.001). This provides compelling evidence that the superiority of our approach is not due to random chance.

Taken together, these results validate the effectiveness of our integrated style-transfer and feedback mechanisms for improving cross-domain generalization in agricultural vision tasks under challenging field conditions.

### 3.4 Ablation study

To validate the contribution of each proposed component, we performed a systematic ablation study using DeepLabv3+ as the backbone. Results are summarized in **Table 3**.

**Table 3:** Evaluation index scores of the ablation study.

Adain	DGB	RAIN	mIoU (%)	Prec. (%)	Rec. (%)	F1 (%)
×	×	×	11.87	24.19	13.62	17.43
✓	×	×	8.97	26.81	10.57	15.16
×	×	✓	36.60	67.42	70.51	68.93
×	✓	×	27.87	58.82	60.80	59.79
×	✓	✓	**39.66**	**71.77**	**74.08**	**72.91**

The best results are highlighted in bold.

To validate the efficacy of each proposed component, we conducted a systematic ablation study using the Deeplabv3+ framework as the backbone. As shown in **Table 3**, the baseline model without any style adaptation modules achieved only 11.87% mIoU, with limited precision (24.19%), recall (13.62%), and F1-score (17.43%), demonstrating poor generalization capability to field subset.

The incorporation of the RAIN module alone significantly improved performance, boosting mIoU to 36.60% and substantially enhancing precision (67.42%), recall (70.51%), and F1-score (68.93%). This demonstrates RAIN’s effectiveness in aligning feature distributions between laboratory and field subset. Similarly, adding only the DGB module also brought notable improvements, achieving 27.87% mIoU with 58.82% precision and 60.80% recall, indicating its contribution to enhancing feature robustness.

The complete proposed framework, integrating both RAIN and DGB modules, achieved the best performance across all metrics: 39.66% mIoU, 71.77% precision, 74.08% recall, and 72.91% F1-score. These results clearly demonstrate the synergistic effect of combining RAIN module with DGB module, highlighting the importance of both components for achieving robustness and generalization.

### 3.5 Activation dynamics and architectural mechanism correlation

**Figure 11:**
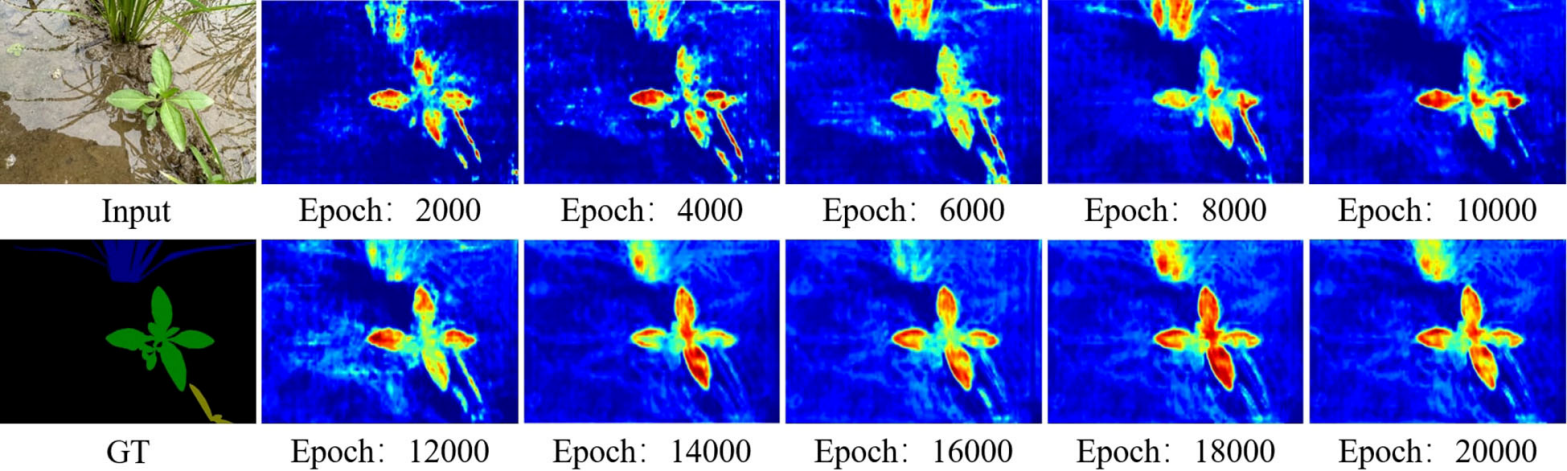
Visualization of feature maps during training epochs.

The Gradient-weighted Class Activation Mapping (Grad-CAM) analysis Selvaraju et al. (2017) visualization illustrates how the model’s attention evolves during training, as shown in **Figure 11**.

In early training stages, Grad-CAM activations are spatially dispersed, with significant responses across background soil and only partial coverage of weed regions. This indicates that the model has not yet learned to fully distinguish between relevant plant structures and irrelevant environmental features. As training proceeds, the activation maps gradually become more concentrated on the actual weed regions. This shift corresponds to improved alignment between style-invariant content features and domain-adaptive representations, supported by the RAIN module and the dynamic gradient mechanism. By the later stages of training, the activations stabilize, focusing more consistently on vegetation areas while reducing spurious responses to non-plant regions.

These observations are consistent with the quantitative improvements in mIoU reported in **Table 3**, where the model achieves 39.66% mIoU after full training. The progressive concentration of attention underscores the role of feedback mechanisms—such as style-augmented alignment and gradient-guided suppression of noise—in helping the model learn robust and localized feature representations for weed segmentation.

### 3.6 Discussion

The experimental results demonstrate that the proposed SWIS framework effectively addresses the challenge of generalizing weed segmentation from controlled laboratory conditions to complex paddy field environments. Our approach achieves a mIoU of 39.66% on the field subset, representing a 58.3% improvement over the best-performing baseline method (DSCA-PSPNet at 25.04%). This performance enhancement can be attributed to the synergistic operation of the two core components: the RAIN module and the DGB module.

The experimental results demonstrate that the proposed SWIS framework substantially narrows the gap between controlled laboratory conditions and complex paddy-field environments for weed segmentation. On the field subset our method attains mIoU=39.66%, corresponding to a ≈58.3% relative improvement over the best baseline (DSCA-PSPNet, 25.04%). This gain stems from the complementary operation of the two core components: the RAIN module and the DGB module.

The ablation results quantify the individual contributions of these modules. RAIN alone increases mIoU from the baseline 11.87% to 36.60% by aligning feature distributions between laboratory and field data via its style-encoding and transformation pipeline; this effect is visually supported by the style-transfer examples in **Figure 6**. DGB alone raises mIoU to 27.87%, confirming that adaptive, adversarial perturbations expand the explored feature manifold and improve robustness. Integrating both modules yields the best outcome (mIoU=39.66%), indicating that RAIN’s macroscopic style alignment and DGB’s microscopic, loss-driven perturbations act synergistically rather than redundantly.

Beyond mIoU, the full model achieves a balanced precision–recall profile (Precision = 71.77%, Recall = 74.08%), avoiding the pronounced precision–recall trade-offs observed in some competitors (e.g., DSCA-PSPNet: Prec. = 93.37%, Rec. = 42.86%). Grad-CAM analysis provides mechanistic insight into these gains: attention maps evolve from scattered activations to focused responses on weed regions as training progresses, mirroring the quantitative improvements shown in the training curve. The model’s convergence around 14,000 epochs and its subsequent stability further attest to the effectiveness of the joint optimization strategy.

In terms of computational efficiency, our model demonstrates a balanced profile suitable for practical deployment. With 496.03 GFLOPs and 49.29 million parameters, it maintains a reasonable computational footprint while delivering robust performance. The inference latency of 66.29 ms (15.09 FPS) for 640×480 inputs enables near real-time processing, which is crucial for field applications requiring prompt responses. Memory requirements are also manageable, with peak usage of 0.57 GB (0.18 GB parameters, 0.38 GB activations), allowing deployment on standard GPU hardware. However, the current computational demands still present certain requirements on the processing capabilities of embedded systems, indicating a clear need for further lightweight optimization in future work to enhance suitability for low-power edge devices.

That said, the proposed model has several limitations that warrant discussion. First, its computational footprint is not yet lightweight enough for real-time deployment on resource-constrained devices, with high parameter counts and inference latency posing challenges for field applications. Second, the overall mIoU remains constrained by the difficulty of accurately segmenting small and rare weed species with limited training samples. This discrepancy not only highlights a key limitation of the current approach but also points to an important direction for future work: developing strategies specifically aimed at detecting and segmenting small or rare weed instances.

When comparing domain robustness, our method shows the most consistent performance across laboratory and field environments: it suffers a smaller relative drop in mIoU (≈56.2%) between domains compared with larger drops (65.8%–74.3%) observed for other methods. This relative robustness highlights the practical value of combining explicit style transformation with adversarial feature exploration for agricultural-vision tasks deployed in heterogeneous, real-world settings.

In summary, the experiments validate the central hypotheses of this work: (1) explicit style transformation via RAIN substantially reduces the laboratory–field domain gap; (2) DGB’s gradient-based perturbations strengthen feature robustness; and (3) the integration of these modules produces a synergistic effect that meaningfully improves cross-domain generalization. Future work should target remaining optical artifacts (e.g., specular reflections) and explore extensions such as physics-aware style modeling or task-specific synthetic augmentation to further close the gap to field-perfect performance.

## 4 Limitations and future work

Despite the promising results achieved by the SWIS framework, several limitations remain. While effective in transferring style features such as soil color and illumination, the current approach struggles with certain physical optical phenomena—most notably specular reflections on water surfaces in paddy fields—which are often misinterpreted as salient features, leading to localized segmentation errors. Moreover, although SWIS exhibits markedly greater robustness than baseline models, a substantial performance gap persists between laboratory (90.54% mIoU) and field (39.66% mIoU) environments, indicating that the domain shift is not yet fully resolved. In addition, the computational overhead introduced by the RAIN and DGB modules, though acceptable for offline processing, poses challenges for real-time deployment on resource-limited agricultural machinery.

The relatively lower mIoU (39.66%) compared to Weed IoU (70.49%) primarily stems from challenges in accurately segmenting small and rare weed species with limited training samples. These minority classes contribute disproportionately to the mIoU calculation due to class-wise averaging, despite their minimal impact on overall weeding effectiveness. This discrepancy highlights an important area for future research: developing specialized strategies for small weed detection.

Future research will focus on three directions. First, integrating polarization cues or explicit reflection models into the style-transfer process to better handle specular artifacts. Second, exploring self-supervised and semi-supervised learning paradigms that can exploit large-scale unlabeled field data to further reduce the laboratory–field performance gap. Third, developing lightweight variants of the RAIN and DGB modules via techniques such as knowledge distillation or neural architecture search, thereby enabling efficient real-time inference on embedded systems for practical in-field deployment. Fourth, devising strategies to address class imbalance and improve the detection of small or rare weed species, potentially through targeted data augmentation or small-sample learning techniques.

## 5 Conclusion

This study addressed the critical challenge of poor cross-domain generalization in deep learning–based weed segmentation, particularly when transferring from controlled laboratory conditions to complex paddy-field environments. To mitigate this domain gap, we introduced the SWIS framework, which combines style transfer and adversarial feature learning to enhance model adaptability. The framework integrates two key components: (1) the RAIN module, which aligns feature distributions through stochastic style transformation, and (2) the DGB module, which improves feature robustness through adaptive adversarial perturbations. Together, these modules enable more reliable segmentation across diverse field conditions.

Extensive experiments demonstrated that SWIS achieves state-of-the-art performance, with 90.54% mIoU on laboratory data and 39.66% mIoU on unseen field data— a 58.3% improvement over the best baseline. Visual analyses further confirmed the model’s enhanced focus on weed regions and reduced sensitivity to environmental noise, though specular reflections remain a challenge. Overall, this work provides a practical and effective solution for robust weed segmentation in real-world agricultural settings. Future extensions will target broader weed species coverage and lightweight optimizations for real-time deployment.

## Data Availability

The original contributions presented in the study are included in the article/Supplementary Material. Further inquiries can be directed to the corresponding authors.
